# Shifts in Geographic Distribution and Antimicrobial Resistance during a Prolonged Typhoid Fever Outbreak — Bundibugyo and Kasese Districts, Uganda, 2009–2011

**DOI:** 10.1371/journal.pntd.0002726

**Published:** 2014-03-06

**Authors:** Maroya Spalding Walters, Janell Routh, Matthew Mikoleit, Samuel Kadivane, Caroline Ouma, Denis Mubiru, Ben Mbusa, Amos Murangi, Emmanuel Ejoku, Absalom Rwantangle, Uziah Kule, John Lule, Nancy Garrett, Jessica Halpin, Nikki Maxwell, Atek Kagirita, Fred Mulabya, Issa Makumbi, Molly Freeman, Kevin Joyce, Vince Hill, Robert Downing, Eric Mintz

**Affiliations:** 1 Division of Foodborne, Waterborne, and Environmental Diseases, Centers for Disease Control and Prevention, Atlanta, Georgia, United States of America; 2 Epidemic Intelligence Service Officer, Centers for Disease Control and Prevention, Atlanta, Georgia, United States of America; 3 Kenya Field Epidemiology Training Program, Nairobi, Kenya; 4 CDC-Kenya, Kisumu, Kenya; 5 Uganda Central Public Health Laboratory, Kampala, Uganda; 6 Bundibugyo District Health Office, Bundibugyo, Uganda; 7 Kasese District Health Office, Kasese, Uganda; 8 Bundibugyo Hospital, Bundibugyo, Uganda; 9 Kagando Hospital, Kagando, Uganda; 10 St. Paul's Health Centre, Kasese, Uganda; 11 CDC-Uganda, Entebbe, Uganda; 12 Uganda Ministry of Health, Kampala, Uganda; Massachusetts General Hospital, United States of America

## Abstract

**Background:**

*Salmonella enterica* serovar Typhi is transmitted by fecally contaminated food and water and causes approximately 22 million typhoid fever infections worldwide each year. Most cases occur in developing countries, where approximately 4% of patients develop intestinal perforation (IP). In Kasese District, Uganda, a typhoid fever outbreak notable for a high IP rate began in 2008. We report that this outbreak continued through 2011, when it spread to the neighboring district of Bundibugyo.

**Methodology/Principal Findings:**

A suspected typhoid fever case was defined as IP or symptoms of fever, abdominal pain, and ≥1 of the following: gastrointestinal disruptions, body weakness, joint pain, headache, clinically suspected IP, or non-responsiveness to antimalarial medications. Cases were identified retrospectively via medical record reviews and prospectively through laboratory-enhanced case finding. Among Kasese residents, 709 cases were identified from August 1, 2009–December 31, 2011; of these, 149 were identified during the prospective period beginning November 1, 2011. Among Bundibugyo residents, 333 cases were identified from January 1–December 31, 2011, including 128 cases identified during the prospective period beginning October 28, 2011. IP was reported for 507 (82%) and 59 (20%) of Kasese and Bundibugyo cases, respectively. Blood and stool cultures performed for 154 patients during the prospective period yielded isolates from 24 (16%) patients. Three pulsed-field gel electrophoresis pattern combinations, including one observed in a Kasese isolate in 2009, were shared among Kasese and Bundibugyo isolates. Antimicrobial susceptibility was assessed for 18 isolates; among these 15 (83%) were multidrug-resistant (MDR), compared to 5% of 2009 isolates.

**Conclusions/Significance:**

Molecular and epidemiological evidence suggest that during a prolonged outbreak, typhoid spread from Kasese to Bundibugyo. MDR strains became prevalent. Lasting interventions, such as typhoid vaccination and improvements in drinking water infrastructure, should be considered to minimize the risk of prolonged outbreaks in the future.

## Author Summary

Typhoid fever is an acute febrile illness caused by the bacteria *Salmonella* Typhi and transmitted through food and water contaminated with the feces of typhoid fever patients or carriers. We investigated typhoid fever outbreaks in two neighboring Ugandan districts, Kasese and Bundibugyo, where typhoid fever outbreaks began in 2008 and 2011, respectively. In Kasese from August 2009–December 2011, we documented 709 cases of typhoid fever. In Bundibugyo from January–December 2011, we documented 333 cases. *Salmonella* Typhi from Bundibugyo and Kasese had indistinguishable molecular fingerprints; laboratory and epidemiological evidence indicate that the outbreak spread from Kasese to Bundibugyo. *Salmonella* Typhi isolated during our investigation were resistant to more antibiotics than isolates obtained from Kasese in 2009. Drinking water in both districts was fecally contaminated and the likely vehicle for the outbreaks. Our investigation highlights that in unchecked typhoid fever outbreaks, illness can become geographically dispersed and outbreak strains can become increasingly resistant to antibiotics. Lasting interventions, including investments in drinking water infrastructure and typhoid vaccination, are needed to control these outbreaks and prevent future outbreaks.

## Introduction

*Salmonella enterica* serovar Typhi (*Salmonella* Typhi) is the Gram-negative bacillus that causes typhoid fever, a systemic infection transmitted through food and water contaminated with human feces. Typhoid fever is characterized by numerous non-specific symptoms, including high fever, headache, malaise, joint pain, abdominal pain, and gastrointestinal symptoms such as nausea, vomiting, constipation, and diarrhea. The case fatality rate is less than 1% with prompt and effective antimicrobial treatment, but may reach 41% in developing countries where access to care is limited [1]. The most serious complication, intestinal perforation, occurs in approximately 3.8% of patients in the developing world; in these areas, reported rates of intestinal perforation range from 0.1–39% [2]. Intestinal perforation has been associated with male gender, older age, delayed or inappropriate antimicrobial therapy, and short duration of symptoms [1], [3], [4].

Typhoid is endemic in many countries with poor sanitation and hygiene and limited access to safe water. Although well-studied in South and Southeast Asia, where it is widely endemic, typhoid fever has only recently been recognized as a significant contributor to the burden of febrile illness in sub-Saharan Africa. Since 2008, severe typhoid fever outbreaks have been reported in rural Malawi [5] and Uganda [6], and in the capital cities of Zimbabwe [7] and Zambia [8]. Typhoid was recently shown to be highly endemic in an urban population in Kenya; the typhoid incidence of 247 cases per 100,000 persons in this area was similar to that found in highly endemic areas in Southeast Asia [9]. Typhoid diagnosis in malaria endemic areas of sub-Saharan Africa is challenged by the similar clinical presentations of typhoid and malaria and the limited availability of laboratory resources in many countries. Blood culture is time and resource intensive, and rapid diagnostic tests, such as TUBEX-TF, are useful for preliminary assessment of potential outbreaks but do not have adequate sensitivity and specificity for diagnosis of individual patients [10]–[12]. Limited data from Africa suggests that antimicrobial resistance is increasing among *Salmonella* Typhi isolates, including a rise in the prevalence of multidrug resistance (MDR), defined as resistance to the three traditional first-line antimicrobials, ampicillin, chloramphenicol, and trimethoprim-sulfamethoxazole [13], and emergence of decreased susceptibility to ciprofloxacin [5], [9], [14].

**Figure 1:**
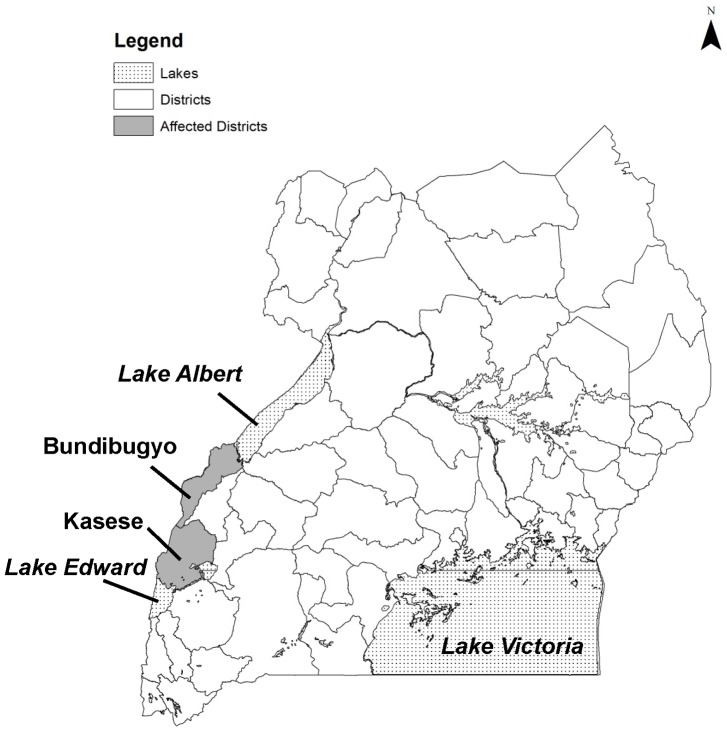
Map of Uganda showing affected districts.

Kasese and Bundibugyo are neighboring rural agricultural districts in western Uganda that border the Democratic Republic of the Congo (**Figure 1**). Epidemics of waterborne and foodborne diarrheal diseases, such as cholera [15] and typhoid fever [6], have historically plagued both districts. Malaria is endemic. Water treatment is largely an individual responsibility, as coverage with improved water sources is low, and neither district has municipal water systems that deliver chlorinated water. An outbreak of typhoid fever with a high rate of intestinal perforation began in 2008 in Kasese district [6]. Among 21 *Salmonella* Typhi isolates obtained through surveillance conducted from March 4 to July 31, 2009, only 1 (5%) was multidrug-resistant and no resistance to nalidixic acid or ciprofloxacin was detected [6].

We report the continuation of the outbreak in Kasese district, present evidence for its extension into Bundibugyo, a neighboring district, and describe a dramatic increase in the prevalence and extent of antimicrobial resistance since 2009. In August 2011, an outbreak of undiagnosed febrile illness, which we later confirmed as typhoid fever, began in the neighboring district of Bundibugyo, and a high number of patients with intestinal perforation were noted in Kasese. In response to a request to investigate from the Uganda Ministry of Health and in collaboration with the Uganda Ministry of Health and Kasese and Bundibugyo District Health Offices, we implemented laboratory-enhanced prospective case finding, conducted retrospective case finding for typhoid fever via medical record reviews, and tested drinking water sources in both districts to determine the scope and likely vehicles of the outbreaks. Outbreak strains were characterized by molecular subtyping and antimicrobial susceptibility testing.

## Materials and Methods

### Case definition

A suspected case of typhoid fever was defined as surgically diagnosed intestinal perforation consistent with *Salmonella* Typhi infection or illness characterized by fever and abdominal pain for ≥1 day and at least one of the following signs or symptoms — gastrointestinal disruptions, such as vomiting, diarrhea, or constipation, general body weakness, joint pain, headache, clinical suspicion for intestinal perforation, or failure to respond to antimalarial medications — with onset from January 1 to December 31, 2011 in Bundibugyo residents and from August 1, 2009 to December 31, 2011 in residents of other districts, including Kasese.

### Case finding

The source populations for identifying cases of typhoid fever in Kasese and Bundibugyo districts were persons seeking care at government-affiliated and private not-for-profit health facilities in these districts and at Fort Portal Regional Referral Hospital in Kabarole district, which borders Kasese and Bundibugyo districts. Cases of surgically-diagnosed intestinal perforation were identified retrospectively from operating room logbooks and chart abstractions of intestinal perforation patients at Kilembe Mines Hospital, Kagando Hospital, Bwera Hospital, St. Paul's Health Center IV, Bundibugyo Hospital, and Fort Portal Regional Referral Hospital. Additional cases were identified retrospectively through linelists of patients with surgically-diagnosed intestinal perforation maintained by Kasese hospitals, and linelists of suspected typhoid fever cases maintained since August 2011 by the District Health Office in Bundibugyo. Beginning in October 2011, typhoid cases were identified prospectively through patient and caregiver interviews in health care facilities and highly affected communities, and through laboratory-enhanced case finding.

### Laboratory-enhanced prospective case finding

Systematic, laboratory-enhanced prospective case finding was conducted in Kasese from November 1 to December 31, 2011 and in Bundibugyo from October 28 to December 31, 2011. Eighty-two health facilities in Kasese and 21 in Bundibugyo were provided with case report forms eliciting information about clinical history, potential risk factors, and socioeconomic status. Health facilities with the capacity to collect specimens in Kasese (15) and Bundibugyo (3) were asked to collect blood and stool from all patients with suspected intestinal perforation and from the first two (Kasese) or three (Bundibugyo) patients who presented at the facility and met the case definition each day.

### Laboratory testing

Blood, serum, and stool were collected according to the above criteria at each facility daily from October 28 to December 31, 2011; prior to this, from October 18 to October 28, 2011, specimens were collected from a sample of clinically-suspected typhoid patients. Supplies for specimen collection and testing were provided by the US Centers for Disease Control and Prevention (CDC). CDC, the Kenya Medical Research Institute (KEMRI), and the Uganda Central Public Health Laboratory (CPHL) trained local laboratory technicians in microbiologic and serologic techniques for typhoid fever diagnosis at one hospital and one upper level health facility in Kasese and at one hospital in Bundibugyo.

Blood (persons 10 years of age and older: 10 ml; children <10 years: 1 ml of blood per year of age) was collected using standard methods. Eight ml of blood (patients ≥10 years) or one-half the sample volume (children <10 years) were inoculated into an Oxoid signal blood culture bottle; the remaining blood was placed in a serum separator tube. Stool was collected according to standard guidelines and inoculated into Cary-Blair transport medium [16]. All samples were held at ambient temperature and transported to district referral laboratories within 72 hours of collection. Blood and stool cultures were performed per standard protocols for isolation of *Salmonella* Typhi [17], [18]. Isolates that were biochemically or serologically typical of *Salmonella* Typhi were forwarded to CPHL for confirmation and to CDC-Atlanta for further characterization, including serotyping, pulsed-field gel electrophoresis (PFGE), and antimicrobial susceptibility testing (AST). PFGE, which is the current gold standard subtyping technique for *Salmonella*, was conducted per standard protocols [19] and PFGE patterns were analyzed using BioNumerics software version 5.01 (Applied Maths, Inc., Austin, TX, USA). AST was performed using disk diffusion [20] and broth microdilution (Sensititre; Trek Diagnostics) according to the manufacturer's instructions. Where applicable, Clinical and Laboratory Standards Institute (CLSI) 2012 interpretive criteria were used to categorize antimicrobial susceptibility results [20]. For drugs that lack CLSI interpretive criteria, results were classified using interpretive criteria from the CDC National Antimicrobial Resistance Monitoring System [21]. Serological testing was performed with TUBEX- TF (IDL Biotech) per product insert.

### Environmental sampling

Water samples collected from drinking water sources in Kasese and Bundibugyo were tested for the presence of total coliforms and *Escherichia coli* in 100 ml, 1 ml, or 100 µl of water using the Colilert-18 test kit (IDEXX, Westbrook, ME). One-ml samples and 100-µl samples were diluted in 100 ml of sterile, distilled water before addition of the Colilert reagent. In Kasese, two surface water sources and six drinking water taps were sampled. In Bundibugyo, river water upstream of a gravity flow scheme (GFS) intake, the GFS outflow tank, two GFS taps, and one tap from a town municipal water supply were sampled.

### Data analysis

Data were entered into electronic databases and analyzed using SAS 9.3 (SAS Institute, Cary, NC). Statistical testing was done using the Fisher's exact test for categorical data and the Wilcoxon rank-sum test for continuous data. For analysis of prospectively-identified cases, non-yes responses for symptoms, animal ownership, and assets were structurally missing on most forms and were imputed as negative responses. *P* values<0.05 were considered significant.

### Ethics statement

The primary purpose of this activity was to identify, characterize, and control disease in response to an immediate public health threat. As such, the human subjects research designee in the Division of Foodborne, Waterborne, and Environmental Diseases at CDC determined that the activities constituted public health response rather than research. Patients with suspected typhoid fever were offered diagnostic testing through routine culture of stool and blood specimens and through serologic testing as part of standard clinical care, and informed consent specifically for this testing was not obtained.

## Results

### Case finding

**Figure 2:**
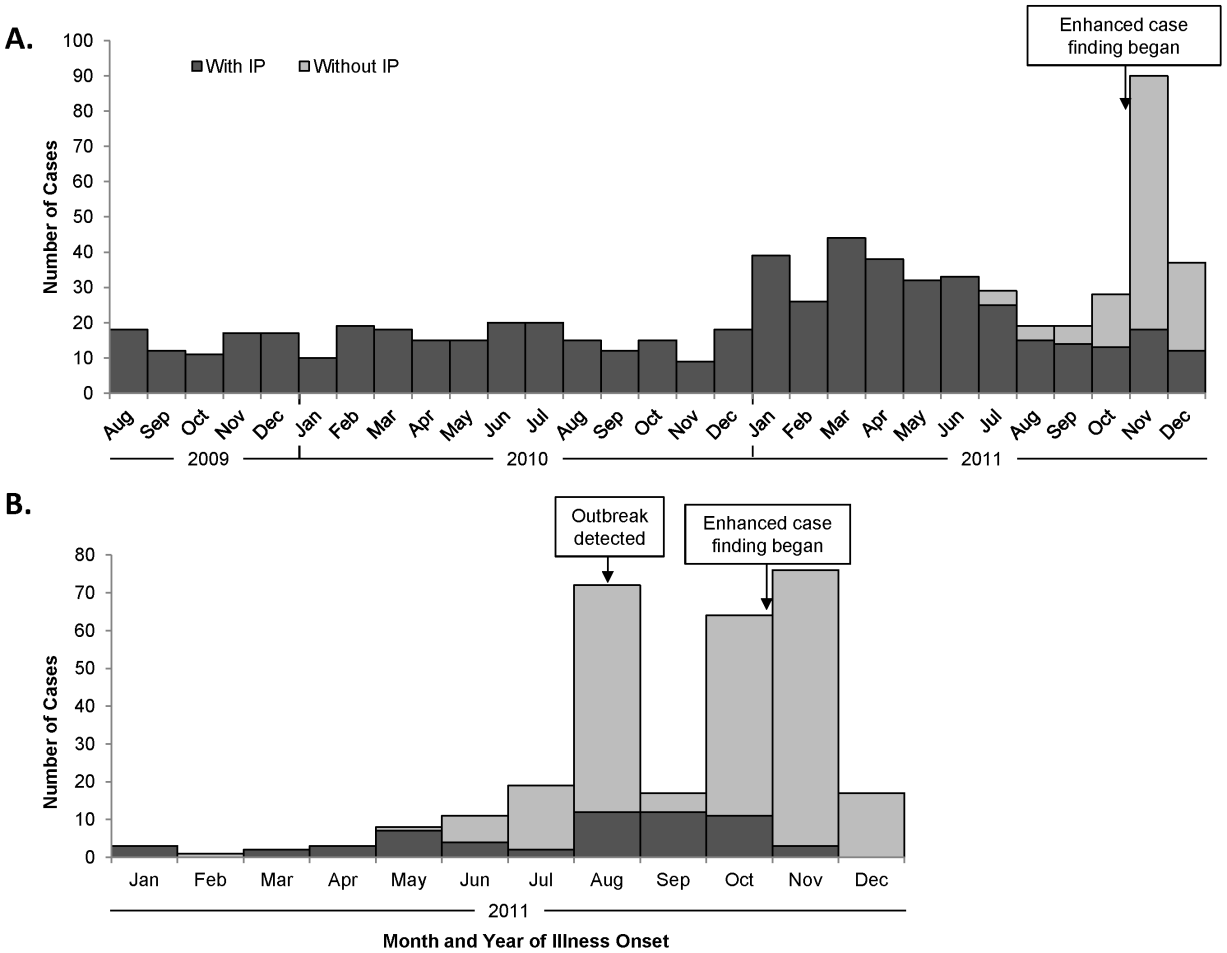
Typhoid fever cases by month of illness onset and intestinal perforation status. (A) Kasese District, August 1, 2009–December 31, 2011, n = 695 with known or estimated onset date and known intestinal perforation status (B) Bundibugyo District, January 1–December 31, 2011, n = 293 with known or estimated onset date and known intestinal perforation status.

During the period August 1, 2009 to January 6, 2012, 1,341 suspected typhoid fever cases were identified, including 1,049 (78%) identified retrospectively and 292 (22%) identified prospectively. Among 1,165 patients for whom district of residence was reported, 709 (61%) resided in Kasese, 333 (29%) resided in Bundibugyo, and 83 (7%) resided in Kabarole district. Thirty-seven were from other districts and three were residents of the Democratic Republic of Congo. Among Kasese patients, more cases with intestinal perforation were recorded in 2011 compared to previous years (**Figure 2A**); during the period of laboratory-enhanced case-finding (November to December 2011), cases with intestinal perforation represented a small fraction of all cases identified. In Bundibugyo, a sharp increase in cases of typhoid fever with and without intestinal perforation was observed beginning in August 2011 (**Figure 2B**); the outbreak was reported to the Uganda Ministry of Health later that month.

**Figure 3:**
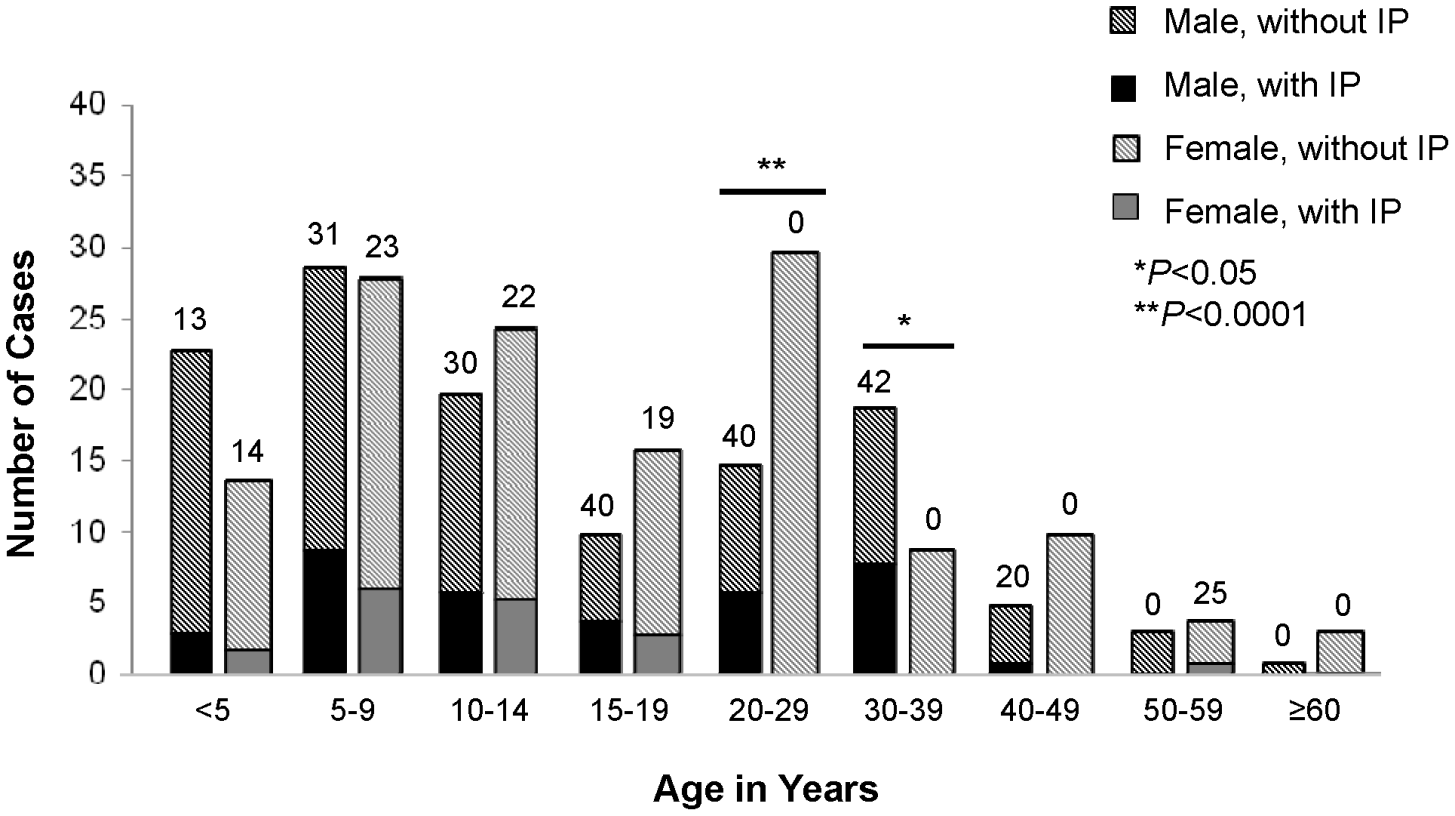
Cases of typhoid fever in Bundibugyo, by age, gender, and intestinal perforation status. January 1–December 31, 2011, n = 244 with known age, sex, and intestinal perforation status, *P = 0.03 and **P = 0.0006.

Intestinal perforation status was recorded or imputed for 697 (98%) of 709 Kasese patients and 293 (88%) of 333 Bundibugyo patients; the frequency of intestinal perforation was 82% and 20%, respectively (**Table 1**). In Kasese, but not Bundibugyo, all sources of retrospective case finding recorded only patients with intestinal perforation instead of all patients with suspected typhoid. Males were disproportionately affected by intestinal perforation in both districts, accounting for 59% and 66% of Kasese and Bundibugyo patients with intestinal perforation, and only 46% and 40% of patients without intestinal perforation, respectively. The median age of Bundibugyo patients was 13 years (range: <1–68 years). Among Bundibugyo patients with intestinal perforation, the median age of males, 15 years, was older than that of females, who had a median age of 10 years (*P* = 0.03). Female patients with intestinal perforation were younger than females without intestinal perforation, who had a median age of 18 years (*P* = 0.008). Among Bundibugyo patients, the proportion with intestinal perforation was significantly higher for males than females for patients aged 20–29 years (40% vs. 0%, *P* = 0.0006) and 30–39 years (42% vs. 0%, *P* = 0.03) (**Figure 3**). Similar associations between age, gender and intestinal perforation status were also identified in Kasese patients.

**Table 1:** Demographic characteristics of patients with suspected or confirmed typhoid fever, Kasese and Bundibugyo districts, Uganda, August 1, 2009–December 31, 2011.

	Kasese				Bundibugyo			
Characteristic	Intestinal Perforation (n = 570†)	No Intestinal Perforation (n = 127†)	All Patients* (n = 709†)	*P* ‡	Intestinal Perforation (n = 59†)	No Intestinal Perforation (n = 234†)	All Patients** (n = 333†)	*P* ‡‡
**Median age (range), in years**								
All patients	17 (<1–85)	15.5 (1–70)	17 (<1–85)		12 (3–50)	13 (1–68)	13 (<1–68)	
Females only^a^	14 (<1–85)	16 (1–70)	15 (<1–85)		10 (3–50)	18 (1–65)	16 (1–65)	0.008
Males only^a^	19 (2–80)	13 (2–70)	18 (2–80)	0.02	15 (3–41)	12 (2–68)	12.5 (<1–68)	
**Gender, Number (%)**								
Male	336 (59)	55 (46)	391 (57)	0.01	39 (66)	88 (40)	142 (45)	<0.0001

*Includes 12 patients for whom intestinal perforation status was reported by clinician as “don't know”.

**Includes 7 patients for whom intestinal perforation status was reported by clinician as “don't know” and 33 patients for whom intestinal perforation status could not be determined from district linelist.

† For some items, n may vary by small numbers due to “don't know” or missing responses.

‡ Wilcoxon rank-sum test (median age) or Fisher's exact test (gender) for difference between Kasese patients with and without intestinal perforation.

‡‡ Wilcoxon rank-sum test (median age) or Fisher's exact test (gender) for difference between Bundibugyo patients with and without intestinal perforation.

a Median age different by gender among all Kasese patients (*P* = 0.002) and among patients with intestinal perforation from Kasese (*P* = 0.0004) and Bundibugyo (*P* = 0.03). Median age did not differ significantly by gender among patients without intestinal perforation in either district.

Kasese patients resided in all 21 sub-counties in the district. During the 29-month case finding period, there were 98 cases of clinically-diagnosed typhoid fever per 100,000 persons district-wide; in 2011 alone, there were 58 cases per 100,000 persons. The Bukonzo West health subdistrict, comprising the western sub-counties of Bwera, Ihandiro, Isango, Karambi, and Mpondwe-Lhubiriha Town Council, had the highest typhoid incidence from August 2009 to December 2011, 115 cases per 100,000 persons. The incidence of typhoid fever was 139 per 100,000 in Bundibugyo District for the period January 1, 2011 to December 31, 2011. During this period, four neighboring Bundibugyo sub-counties had an incidence of typhoid fever greater than 100 cases per 100,000 persons: Kirumya (990 per 100,000), Bubukwanga (234 per 100,000), Bukonzo (199 per 100,000), and Bundibugyo Town Council (155 per 100,000).

### Laboratory-enhanced case finding

Case report forms for 149 Kasese residents, 128 Bundibugyo residents, and 13 residents of other districts or for whom district of residence could not be determined were completed at health facilities from October 11, 2011 to January 6, 2012. The most common symptoms of the 277 case-patients from Kasese and Bundibugyo, other than fever and abdominal pain (both required by the case definition), were weakness (84%), headache (82%), and joint pain (71%) (**Table 2**). Six percent of patients had intestinal perforation. Before the visit where the case report form was completed, patients reported feeling ill for a median of 7 days (range 1–240 days, n = 267), including having fever for a median of 6 days (range 1–240 days, n = 268), and abdominal pain for a median of 4 days (range 1–730 days, n = 262).

**Table 2:** Clinical features*of typhoid fever patients reported on case report forms, Kasese and Bundibugyo Districts, April 15, 2011–December 31, 2011.

	No. (%) Kasese Patients (n = 149†)	No. (%) Bundibugyo Patients (n = 128†)	No. (%) All Patients (n = 277†)
**Clinical feature**			
Weakness	130 (87)	104 (81)	234 (84)
Headache	129 (87)	97 (76)	226 (82)
Joint Pain	106 (72)	88 (70)	194 (71)
Diarrhea	61 (41)	50 (39)	111 (40)
Not responding to antimalarials	51 (35)	42 (35)	93 (35)
Vomiting	52 (35)	39 (31)	91 (33)
Constipation	29 (20)	39 (31)	68 (25)
Intestinal Perforation	11 (8)	4 (3)	15 (6)

*Case definition specified that fever and abdominal pain must be present.

† For some items, n may vary by small numbers due to “don't know” responses.

Seventy percent of respondents reported seeking care for their illness before the health care visit during which the case report form was completed (**Table 3**). Patients most frequently sought care at a drug shop or pharmacy (49%) or a health center or hospital (48%); a small fraction consulted an herbalist (7%) or traditional healer (1%). Antibiotics were taken by 45% of patients. Metronidazole, which is not effective against *Salmonella* Typhi, was the antimicrobial most frequently reported and was used by 36% of patients who reported taking antimicrobials. Ciprofloxacin, co-trimoxazole, and amoxicillin were taken by 27%, 26%, and 24% of patients who reported taking antimicrobials, respectively; only 5% reported taking chloramphenicol. Nearly one-quarter (23%) of patients who reported taking antimicrobials had taken ≥2 agents to treat the same illness episode.

**Table 3:** Clinical history of typhoid fever patients, Kasese and Bundibugyo Districts, April 15, 2011–January 6, 2012.

Characteristic	No. (%) Kasese Patients (n = 149†)	No. (%) Bundibugyo Patients (n = 128†)	No. (%) All Patients (n = 277†)
**Previously sought care for this illness**	105 (74)	83 (66)	188 (70)
Sought care from:*	n = 105	n = 83	n = 188
Drug shop/Pharmacy	51 (49)	41 (49)	92 (49)
Health center/Hospital	50 (48)	40 (48)	90 (48)
Herbalist	7 (7)	6 (7)	13 (7)
Traditional healer	0 (0)	1 (1)	1 (1)
Other‡	1 (1)	5 (6)	6 (3)
**Took Antibiotics**	60 (47)	51 (44)	111 (45)
Antibiotics taken:*	n = 60	n = 51	n = 111
Metronidazole	21 (35)	19 (37)	40 (36)
Ciprofloxacin	14 (23)	16 (31)	30 (27)
Co-trimoxazole	17 (28)	12 (24)	29 (26)
Amoxicillin	15 (25)	12 (24)	27 (24)
Chloramphenicol	2 (3)	3 (6)	5 (5)
Other‡‡	7 (12)	4 (8)	11 (10)
≥2 antibiotics	12 (20)	14 (27)	26 (23)
**Took Antimalarials**	74 (56)	83 (70)	157 (62)

† For some items, n may vary by small numbers due to “don't know” responses.

*Percentage totals may be >100%; respondents could select ≥1 source of care and antibiotic.

‡ Kasese patients: 1 school nurse; Bundibugyo patients: 5 friend or relative.

‡‡ Kasese patients: 1 each cephalexin, erythromycin, gentamycin, 4 unknown; Bundibugyo patients: 1 each ceftriaxone and doxycycline, 2 unknown.

In Kasese, tap water was the most commonly reported primary source of drinking water during the month before illness onset and was reported by 84 (62%) of 136 patients. Other primary drinking water sources were stream or river water, spring water, and wells, used by 22 (16%), 14 (10%), and 2 (1%) patients, respectively. In Bundibugyo, tap water was also the most common primary source of drinking water and was reported by 56 (46%) of 122 patients. Forty-three (35%) patients used stream or river water, 11 (9%) used spring water, 5 (4%) used well water, and 2 (2%) used bottled water. Among the 50 Bundibugyo patients who reported tap water as their primary drinking water source and also reported their sub-county of residence, 24 (48%) resided in subcounties where tap water was provided by a single GFS, the Kirumya-Bubukwanga GFS, and 8 (16%) resided in Bundibugyo Town Council, the district's largest town. Among the 35 Bundibugyo patients who used river water as their primary drinking water source and reported the name of the river they used, 15 (43%) used the Kirumya River, which is the source of the Kirumya-Bubukwanga GFS. Clustering by drinking water source was not observed among Kasese patients.

Among 208 patients, 30 (14%) reported treating their water in the month before they became ill. Among those who treated their water, 20 (67%) boiled water, 7 (23%) used a chlorine product, 1 (3%) used PuR, a chlorination-flocculation product, and 2 (0.7%) used a treatment method not listed.

### Intestinal perforation risk factors

Significant differences were observed in the symptoms, clinical histories, and socioeconomic status of the 18 patients with confirmed or suspected intestinal perforation and the 250 patients without intestinal perforation who were identified through prospective laboratory-enhanced case-finding (**Table 4**). Patients with intestinal perforation were more likely than those without intestinal perforation to have sought health care for the same illness episode before the visit when the enrollment form was completed (100% vs. 69%, *P* = 0.004) and to report that care was sought at a health center or hospital (88% vs. 46%, *P* = 0.0008). Patients with intestinal perforation reported not responding to antimalarials more frequently than patients without intestinal perforation (71% vs. 31%, respectively; *P* = 0.003), although reported antimalarial use did not differ by intestinal perforation status. Patients with intestinal perforation were more likely than those without intestinal perforation to report taking antibiotics (75% vs. 44%, respectively, *P* = 0.02), and among patients who took antibiotics, were more likely to report taking chloramphenicol (25% vs. 1%, respectively; *P* = 0.003). Compared to patients without intestinal perforation, more than twice the proportion of patients with intestinal perforation reported taking ≥2 antibiotics to treat the same illness episode (58% vs. 24%; *P* = 0.04). Patients with intestinal perforation were less likely to own two or more listed household items (radio, mobile telephone, foam mattress, bicycle, and motorcycle) than patients without intestinal perforation (56% vs. 80%, respectively; *P* = 0.03); employment and number of animals owned did not vary by intestinal perforation status.

**Table 4:** Selected characteristics of patients with suspected typhoid fever, by intestinal perforation (IP) status, April 15, 2011–January 6, 2012.

Characteristic	No. (%) With IP (n = 18*)	No. (%) Without IP (n = 250*)	*P* **
**Clinical Characteristics**			
**Previously sought care for this illness**	17 (100)	166 (69)	0.004
Sought care from:†	n = 17	n = 166	
Health center/Hospital	15 (88)	76 (46)	0.0008
Drug shop/Pharmacy	5 (29)	88 (48)	
Herbalist	2 (12)	11 (7)	
**Took antibiotics**	12 (75)	98 (44)	0.02
Antibiotics:	n = 12	n = 98	
Metronidazole	6 (50)	35 (36)	
Co-trimoxazole	1 (8)	29 (30)	
Ciprofloxacin	4 (33)	26 (27)	
Amoxicillin	1 (8)	26 (27)	
Chloramphenicol	3 (25)	1 (1)	0.004
Other‡	3 (25)	9 (9)	
≥2 antibiotics	7 (58)	24 (24)	0.04
**Took antimalarials**	10 (67)	154 (62)	
**Reported not responding to antimalarials**	10 (71)	76 (31)	0.003
**Socioeconomic status**			
Owned ≥2 household items	10 (56)	201 (80)	0.02
Owned ≥2 animals	7 (39)	86 (34)	
Percent unemployed	0 (0)	22 (9)	

*For some items, n may vary by small numbers due to “don't know” responses.

**Fisher's Exact test.

† Percentage totals may be >100%; respondents could select ≥1 source of care and antibiotic.

‡ Patients with intestinal perforation: 1 each ceftriaxone, gentamycin, and unspecified; patients without intestinal perforation: 1 each cephalexin, doxycycline, erythromycin, and gentamycin, and 5 unspecified.

### Laboratory investigation

*Salmonella* Typhi was isolated from seven (9%) of 74 blood cultures and one (2%) of 47 stool cultures from Kasese patients, and from 15 (21%) of 72 blood cultures and one (10%) of 10 stool cultures from Bundibugyo patients. In total, eight (11%) of 75 Kasese patients and 16 (20%) of 79 Bundibugyo patients tested were culture-confirmed. Two (14%) of 14 patients with intestinal perforation who had a blood culture performed had *Salmonella* Typhi isolated.

**Figure 4:**
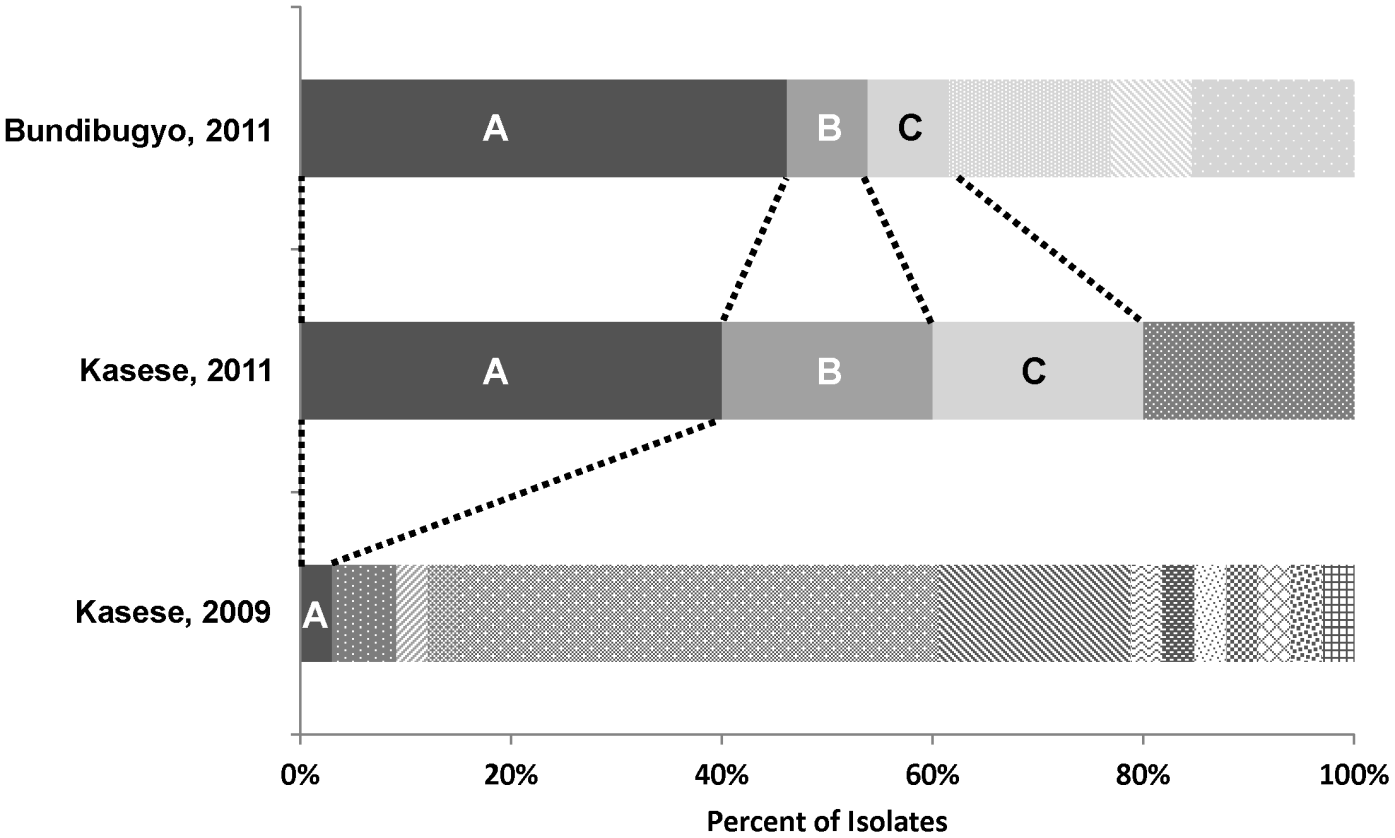
Diversity of PFGE patterns among *Salmonella* Typhi isolated from Kasese and Bundibugyo patients. Each *Xba*I/*Bln*I PFGE pattern combination is represented by a different shading; pattern combinations designated by letter are shared across districts and/or years. *Salmonella* Typhi isolated from October 18–December 31, 2011 were from 13 Bundibugyo and 5 Kasese patients; among these, we observed 6 and 4 pattern combinations, respectively. Investigations in Kasese from March 4–April 17, 2009 yielded 33 *Salmonella* Typhi isolates, among these 13 pattern combinations were identified [6].

*Salmonella* Typhi isolates from five of the eight culture-confirmed Kasese cases and 13 of the 16 culture-confirmed Bundibugyo cases were further characterized at the U.S. Centers for Disease Control and Prevention in Atlanta. PFGE subtyping revealed four distinct *Xba*I/*Bln*I pattern combinations among the five Kasese isolates and six distinct *Xba*I/*Bln*I pattern combinations among the 13 Bundibugyo isolates. When compared to the PulseNet Global *Salmonella* Typhi database, a database of globally distributed Typhi isolates, these pattern combinations were unique with the exception of a single pattern combination, pattern combination A, which was observed in the single chloramphenicol resistant Kasese isolate from 2009 (**Figure 4**). Among the 2011 isolates, pattern combination A was the pattern combination most frequently observed and was shared by two Kasese isolates and six Bundibugyo isolates, all of which were chloramphenicol resistant. Two novel pattern combinations, designated B and C, were also shared by isolates from both districts.

Eighteen isolates were tested for susceptibility to a panel of antimicrobials that included amoxicillin/clavulanic acid, ampicillin, ceftriaxone, chloramphenicol, ciprofloxacin, nalidixic acid, streptomycin, sulfisoxazole, tetracycline, and trimethoprim-sulfamethoxazole. Two isolates, both from Bundibugyo patients, were pan-susceptible. Fifteen (83%) isolates were resistant to ampicillin, chloramphenicol, and trimethoprim-sulfamethoxazole (MDR), and were also resistant to sulfisoxazole, streptomycin, and tetracycline. A single isolate, from Kasese, was resistant to nalidixic acid and showed intermediate susceptibility to ciprofloxacin; it was fully susceptible to other antimicrobials tested.

TUBEX-TF serological testing was positive for 23 (35%) of 65 Kasese patients and 13 (45%) of 29 Bundibugyo patients. Among the 59 patients who had both a blood culture and a TUBEX-TF serological test performed, six (10%) had *Salmonella* Typhi isolated from blood or stool and tested positive by TUBEX-TF, four (7%) had a blood culture positive for *Salmonella* Typhi but a negative TUBEX-TF test, 15 (25%) had blood culture negative for *Salmonella* Typhi but had a positive TUBEX-TF test, and 34 (58%) were blood culture and TUBEX-TF negative. Thirteen (7%) of 182 Kasese and Bundibugyo patients tested were positive for malaria by RDT or blood smear; one (<1%) of 124 patients with blood or stool culture and malaria diagnostic results reported was positive for both typhoid and malaria.

### Environmental sampling

Water samples were collected from six drinking water taps and two surface water sources in Kasese and three drinking water taps and one surface water source in Bundibugyo. Total coliforms were present in all samples. *E. coli* were detected in 100 mL of water from four (67%) of six Kasese drinking water taps (corresponding to a concentration of ≥1 cfu/100 mL) and in 100 µl of water from the rivers Rwimi and Hima (corresponding to a concentration of ≥1000 cfu/100 mL). In Bundibugyo, *E. coli* were present in 100 ml of water from the Kirumya river upstream of the Kirumya-Bubukwanga GFS intake, from the GFS outflow tank, and from two taps on the GFS and one tap in the Bundibugyo Town Council municipal water supply. *E. coli* were also present in 1 ml of water from the two taps on the GFS (corresponding to a concentration of ≥100 cfu/100 mL); the Bundibugyo Town Council municipal water supply and Kasese tap water were not tested at volumes below 100 mL.

## Discussion

A large and severe typhoid fever outbreak in rural western Uganda persisted from 2008 through 2011, spread to a neighboring district, and became more refractory to antimicrobial treatment. In 2009, an investigation suggested that contaminated drinking water was the most likely vehicle of infection, and general prevention measures such as hand washing, improved sanitation, and promotion of household water treatment were recommended [6]. Absent a sustained and widespread intervention campaign, a resurgence of cases with intestinal perforation was investigated in 2011. Molecular subtyping and epidemiologic evidence from the 2011 investigation indicate that the typhoid outbreak persisted in Kasese and spread to the neighboring district of Bundibugyo. Compared to *Salmonella* Typhi isolated from Kasese patients over a six-week period in 2009, of which only one isolate (1/21; 5%) was multidrug resistant [6], isolates obtained from Kasese and Bundibugyo patients over the three-month period October to December 2011 were more likely to be multidrug resistant. Additionally, an isolate with reduced susceptibility to ciprofloxacin, the current recommended first-line treatment for uncomplicated typhoid, was identified for the first time among outbreak strains. Across the 2009 and 2011 enhanced case finding periods, the frequency of co-trimoxazole and chloramphenicol use were similar (29% vs. 26% and 9% vs. 5%, respectively), indicating that changes in antibiotic use do not explain the increased frequency of MDR isolates in 2011. These findings demonstrate that the ramifications of severe, uncontrolled typhoid outbreaks include outbreak strains that become increasingly resistant to lifesaving antibiotics and the spread of disease to neighboring areas.

Selective recognition and documentation of patients with intestinal perforation, the most severe complication of typhoid fever, led to an underestimation of the magnitude of the outbreak and an overestimation of the proportion of reported cases with intestinal perforation. Although the overall proportion of cases with intestinal perforation was 82% in Kasese and 20% in Bundibugyo, prospective case finding in district health facilities showed that patients with intestinal perforation represented only 8% of Kasese patients and 3% of Bundibugyo patients, or only 6% of all typhoid cases. Inflation of the intestinal perforation rate as an artifact of retrospective case finding methods was more pronounced in Kasese, where linelists recorded only patients with intestinal perforation, compared with Bundibugyo, where all suspected typhoid cases were included on the linelist. Extrapolating from the 570 Kasese intestinal perforation cases identified from August 1, 2009 to December 31, 2011 and the 8% intestinal perforation rate observed through prospective case-finding, we estimate that 7,125 cases of typhoid occurred among Kasese residents during this period, giving an estimated annual incidence of 409 cases per 100,000 persons. In Bundibugyo, where 59 cases with intestinal perforation were reported from January 1 to December 31, 2011 and the intestinal perforation rate was 3%, we estimate that there were 1,967 typhoid fever cases and an annual incidence of 820 cases per 100,000 persons. Although based on a different case definition and different case-finding method, these incidences exceed rates observed in African urban slums of 247 per 100,000 [9], and indicate that intense, sustained typhoid transmission occurs in rural areas of sub-Saharan Africa.

Males were disproportionately affected by intestinal perforation; in Bundibugyo, and to a lesser extent in Kasese, this was more pronounced among adults. In both districts, females with intestinal perforation were younger than those without intestinal perforation, and the opposite was observed for male patients. The higher frequency of intestinal perforation in males compared to females has been well-documented in several case-series in Africa [4], [22], [23], Asia [1], [24], and the Caribbean [25], and male sex was identified as a risk factor for intestinal perforation among hospitalized typhoid patients in Turkey [3]. The reasons for this often observed association remain unknown. We found fewer published observations of the influence of age on the association between intestinal perforation status and sex. A single study in South Africa found that typhoid clinical features varied by sex among adults but not children; however, in this study no cases of intestinal perforation occurred among female patients of any age [4]. The differences observed in our study may reflect age and gender-specific care seeking behaviors or treatment adherence. Alternatively, the relatively low rates of intestinal perforation observed in women beyond the age of puberty may indicate that sex hormones play a role in disease pathogenesis; in mouse models of typhoid fever, estrogen decreased the intensity of infection [26].

Certain clinical factors, such as multiple health care visits and taking two or more antibiotics, were associated with intestinal perforation, suggesting that initial treatments were not effective. Inadequate treatment of typhoid was previously described as a risk factor for intestinal perforation among hospitalized typhoid patients [3]. Chloramphenicol was the only antibiotic specifically associated with intestinal perforation, and this may be related to widespread chloramphenicol resistance among outbreak strains. Loss of chloramphenicol susceptibility was previously associated with a high rate of intestinal perforation during a typhoid fever outbreak in Kinshasa, Democratic Republic of Congo, where chloramphenicol was the drug of choice for empiric treatment of typhoid [27]. Unlike in Kinshasa, expansion of chloramphenicol resistance was not associated with a detectable increase in the intestinal perforation rate; this may be because chloramphenicol use was rare among Uganda typhoid patients.

For the first time in this epidemic, MDR *Salmonella* Typhi isolates predominated among the outbreak strains and an isolate resistant to nalidixic acid and with reduced susceptibility to ciprofloxacin was identified. Recently, there have been multiple reports of widespread MDR *Salmonella* Typhi in East and Central Africa. In the Democratic Republic of Congo, 30% of *Salmonella* Typhi isolated from 2007–2011 were MDR, and 15% showed nalidixic acid resistance and decreased susceptibility to ciprofloxacin [14]; in an urban area in Kenya, 78% of *Salmonella* Typhi isolates were MDR and 3% were resistant to nalidixic acid [9]; and in a 2009 outbreak in Malawi, all isolates were MDR and 10% were resistant to nalidixic acid [5]. Suboptimal dosage and duration of therapy, as might occur with poor prescribing practices and poor adherence to therapy, may accelerate the development of antimicrobial resistance. We documented widespread improper antibiotic use among Kasese and Bundibugyo patients; of those who reported taking ciprofloxacin, only 18% completed the recommended 14-day course and half took ciprofloxacin for 5 days or fewer. Development of full ciprofloxacin resistance, alone or in combination with MDR, would further limit treatment options in western Uganda, as there are practical concerns about the use of the three primary alternatives to ciprofloxacin there. The best orally administered alternative to ciprofloxacin, azithromycin, is expensive and not stocked by Ministry of Health-sponsored facilities; another oral therapy, gatifloxacin, has been shown to be effective in areas with widespread nalidixic acid resistance but has been pulled from several markets due to severe side effects in adults and is not licensed in Uganda; the third possibility, ceftriaxone, is stocked at Ugandan hospitals and some health centers but must be administered parenterally. In this resource-limited setting, emergence of ciprofloxacin resistant *Salmonella* Typhi in the absence of alternative oral therapies would be a devastating blow to the typhoid pharmacopeia, and would likely result in increased rates of complications, including intestinal perforation.

Piped drinking water was the probable primary transmission vehicle in both districts. Although we did not isolate *Salmonella* Typhi from water sources, the presence of *E. coli*, a marker of fecal contamination, and the epidemiologic evidence are consistent with a waterborne source of typhoid infection. Despite an ongoing typhoid fever outbreak and recommendations to implement safe water interventions, the percentage of patients who reported treating water in the month before they became ill decreased from 22% in 2009 to 14% in 2011; in both districts the most common reason for not treating water was the belief that treatment was not necessary. This is consistent with the limited uptake of point-of-use water treatment observed among at-risk populations in other parts of the world [28], [29]. Furthermore, uptake in Kasese in 2011 may have been particularly low because the outbreak had passed the acute emergency phase, in which supplies were provided free of charge and treatment could be viewed as a temporary measure. The enduring nature of this typhoid outbreak and emergence of increasingly drug-resistant strains indicate a need for alternative interventions. Providing treated water through the many piped water systems that exist in both districts, and expanding these systems to new areas would provide long-term reductions in risk of typhoid and other waterborne diseases. Typhoid vaccination should be strongly considered as a medium-term intervention that controls the typhoid outbreak while water infrastructure improvements are made.

These findings are subject to several limitations. The absence of systematic typhoid surveillance before implementation of laboratory-enhanced case-finding made it difficult to ascertain the true scope of the outbreak. During enhanced case-finding, cases were missed, both because the system did not capture patients who did not seek care at government or private not-for-profit health facilities and because some facilities chose not to participate in case-finding. Missed cases may have differed from reported cases systematically by sub-county of residence, socioeconomic status, clinical history, and other factors. Typhoid fever is a diagnostic challenge, particularly in malaria endemic areas where a large proportion of fevers are attributed to malaria, and we attempted to balance the sensitivity and specificity of the case definition with this in mind. We included abdominal pain to increase specificity; however, by improving specificity, we likely lost sensitivity to detect typhoid patients early in the clinical course, before abdominal pain develops. This may have impacted blood culture positivity, since positivity is highest in the week following symptom onset [30]. The high number of patients taking amoxicillin, ciprofloxacin, and co-trimoxazole likely biased organisms recovered to those resistant to these therapies. The imputation of missing data as negative responses for clinical signs and symptoms and socioeconomic factors could have biased results towards underestimation of frequencies. For example, had missing data been censured, the calculated intestinal perforation rates would have been higher, 12% and 5% in Kasese and Bundibugyo, respectively. Additionally, due to the low positivity of blood cultures, it is possible that many patients with non-typhoidal febrile illness were included in the case definition, and the true proportion with IP may have been higher. Accurate identification of sub-counties of residence was challenged by missing data, multiple spellings for the same location, and reorganization of several western Kasese sub-counties in 2011.

Comprehensive, laboratory-enhanced surveillance for typhoid fever is necessary in outbreak settings to characterize affected populations, monitor disease trends, target interventions, and assess their impact. Reliance on severe complications, such as intestinal perforation, as surrogates for all cases can be misleading when complication rates vary over time due to changes in treatment practices or antimicrobial resistance. Given the many reports of MDR and nalidixic acid resistant *Salmonella* Typhi in sub-Saharan Africa, improving laboratory diagnostic capacity is crucial for making appropriate, timely treatment recommendations. In areas of high incidence where conventional approaches to safe water have not stopped transmission and drug resistant strains are circulating, novel approaches, such as typhoid vaccination, should be considered.
